# The saga to monitor and control norovirus: the rise of GII.17

**DOI:** 10.1099/jgv.0.002118

**Published:** 2025-06-06

**Authors:** Gabriel I. Parra, Kentaro Tohma, Lauren A. Ford-Siltz, Kelsey A. Pilewski, Joseph A. Kendra

**Affiliations:** 1 Division of Viral Products, Center for Biologics Evaluation and Research, Food and Drug Administration, Silver Spring, MD, USA

**Keywords:** antigenic diversity, diarrhea, histo-blood group antigen (HBGA), norovirus, virus evolution

## Abstract

Norovirus is a major cause of acute gastroenteritis in all age groups, with recent surges of cases in Europe and the USA reinforcing the influence of this virus on human health. Despite its societal impact, no vaccine or antiviral drug is available. The development of these countermeasures has been impaired at least in part by the extreme genetic and antigenic diversity of noroviruses. Here, we reviewed historical norovirus outbreaks, including the pandemics of GII.4 norovirus that were first documented in the mid-1990s, sporadic increases of non-GII.4 norovirus (e.g. GII.17 and GII.2) during the 2010s and, most recently, the ongoing large outbreaks caused by a new cluster of GII.17 noroviruses. This five-decade-long journey of tracking noroviruses in the human population illustrates the importance and challenges of battling this evolving virus.

## Episode I: The Norwalk Menace

The notoriety of norovirus began in 1929 with the publication of the clinical description of ‘winter vomiting disease’ by paediatrician John Zahorsky [1]. This disease was characterized by 2–3 days of explosive vomiting and diarrhoea – often accompanied by abdominal pain, malaise and a low-grade fever – and was mostly associated with young children in the fall and winter seasons. The cause of ‘winter vomiting disease’ continuously stumped general practitioners until 1972, after inoculum from an outbreak in Norwalk, Ohio, was administered to a volunteer. The individual became ill with gastroenteritis shortly following contact with the so-called Norwalk agent, and viral particles were visualized in the stool filtrate [2]. The same method (immune electron microscopy) was later used to identify similar small (~30 nm) viral particles in the stool filtrates from multiple outbreaks of gastroenteritis, including ‘Montgomery agent’ and ‘Hawaii agent’, which caused two small, independent outbreaks in 1971 [3], and ‘Snow Mountain agent’, which caused a large outbreak in a resort camp in 1976 [4]. Additional gastroenteritis outbreaks caused by infectious non-bacterial agents or Norwalk-like agents were described during the 1970s and 1980s, but the lack of tools impeded the full characterization of these viruses [5,11].

Experimental studies on these agents were limited, partially due to the inability to propagate them in cell culture or to infect small animal models [12,14]. Thus, human challenge studies and epidemiological investigations were the definitive approaches to explore their biology and immunity. Human subjects challenged with ‘Norwalk’, ‘Hawaii’ and ‘Montgomery’ agents, for example, did not develop gastroenteritis when rechallenged with the same agent within a few months of the initial infection. However, heterologous challenge with ‘Norwalk’ and ‘Hawaii’ agents still resulted in disease, suggesting that these agents were antigenically distinct. The relationship between the ‘Montgomery agent’ and the other two agents was unclear [15]. Two additional pieces of information supported the notion of antigenic diversity among these agents: (i) the lack of viral particle aggregation (as tested in immune electron microscopy) of the ‘Snow Mountain agent’ with sera from individuals infected with ‘Norwalk’ and ‘Hawaii’ agents [4] and (ii) the lack of protection during two subsequent outbreaks (11 months apart) of acute infectious nonbacterial gastroenteritis at the former Henryton State Hospital of Maryland in 1971 [5,6]. These important studies also hinted that there may be a genetic component to susceptibility, as not all individuals developed gastroenteritis after inoculation [16]. Indeed, over 20 years later, it was demonstrated that humans have a genetic predisposition for infection with norovirus, in which individuals with a functional fucosyltransferase 2 (FUT2) enzyme, known as ‘secretors’, are more susceptible to infections with different noroviruses [17,19]. FUT2 plays a role in the synthesis of certain histo-blood group antigen (HBGA) carbohydrates expressed on the surface of intestinal epithelial cells in the human body, and these carbohydrates have been shown to act as attachment factors that facilitate norovirus infection [20,21].

In 1993, the genomes of the ‘Norwalk agent’ and ‘Southampton agent’ were sequenced and classified into the *Caliciviridae* family of positive-sense RNA viruses [22,23]. Later, in the early 2000s, the International Committee on the Taxonomy of Viruses designated ‘Norwalk agent’ and the other ‘Norwalk-like viruses’ as norovirus [24]. The norovirus genome was demonstrated to be divided into three ORFs; ORF1 encodes the non-structural proteins, including the polymerase, and ORF2 and 3 encode the major (VP1) and minor (VP2) capsid proteins, respectively [22,23]. The sequencing of additional noroviruses facilitated the development of universal primers and better methods of detection, which increased the awareness of the role of norovirus in acute gastroenteritis in humans [25,28]. With the establishment of the classification system, over 40 different genotypes capable of infecting humans were identified, and the historical ‘agents’ responsible for small and large gastroenteritis outbreaks in the 1970s were finally granted nomenclature. ‘Norwalk’, ‘Southampton’, ‘Chiba’, ‘Montgomery’, ‘Hawaii’ and ‘Snow Mountain’ agents became prototypes for GI.1, GI.2, GI.4, GI.5, GII.1 and GII.2 genotypes, respectively [29,30]. Other viruses, like those detected in the Shippensburg outbreak from 1972 and the Toronto outbreak from 1985, were determined to be caused by GII.3 [7,8]. The Otofuke outbreak in 1978 in Hokkaido, Japan, was caused by GI.3 [11], and the two Henryton outbreaks detected in 1971 were caused by GII.2 followed by GII.6 [6]. Thus, a large number of different genotypes were described to cause outbreaks during the early years of norovirus research and discovery.

## Episode II: the attack of GII.4 noroviruses

**Fig. 1.**
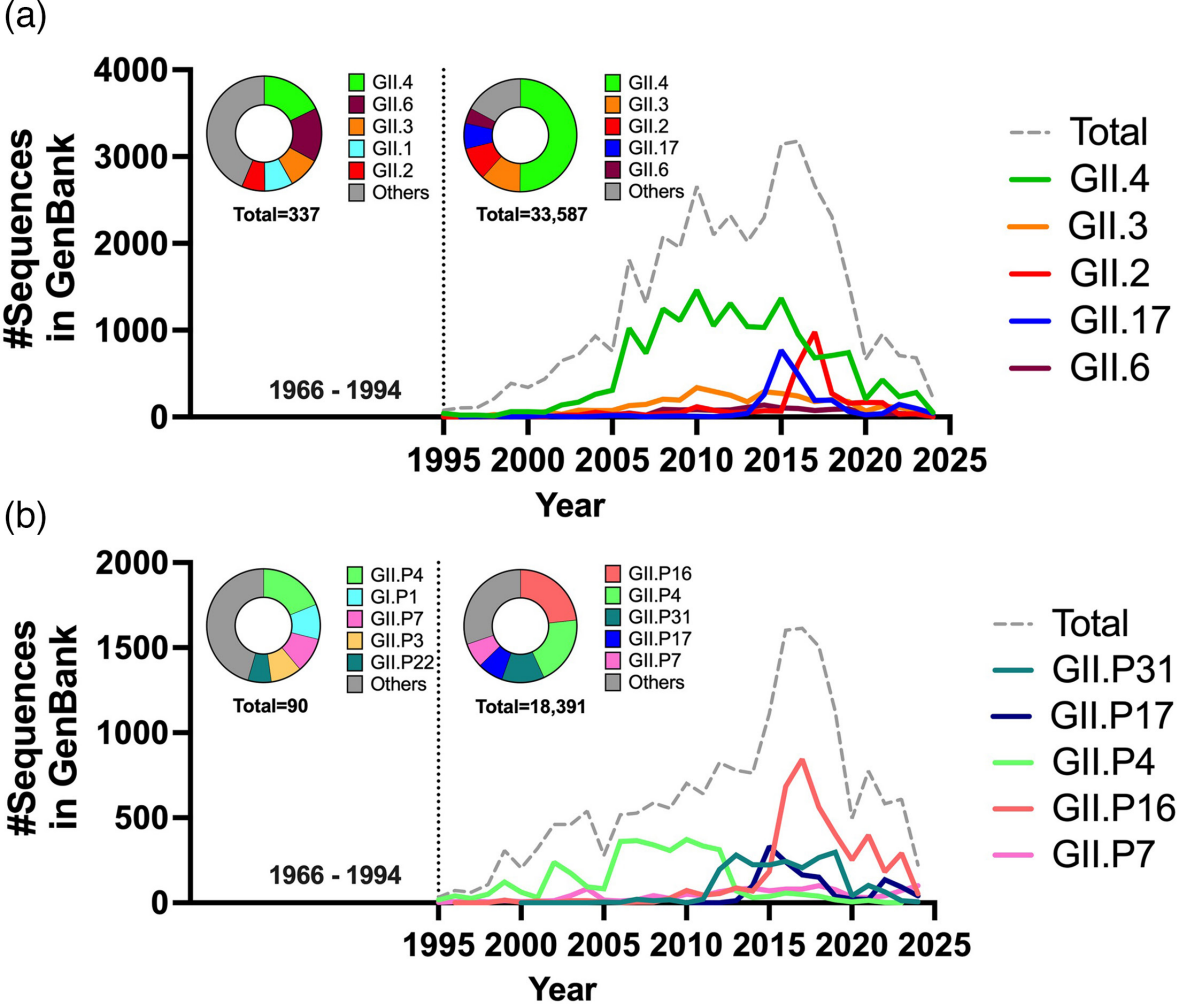
Temporal distribution of major norovirus genotypes and polymerase types. The human norovirus sequences (≥100 nt in length, *n*=39,345) deposited in GenBank were collected (15 January 2025) and annotated by time, location and genotypes using Norovirus Typing Tool when available [110]. (**a**) Distribution of capsid genotypes. The solid-coloured lines denote the temporal trend of the top five major genotypes, GII.4, GII.3, GII.2, GII.17 and GII.6 per year. (**b**) Distribution of polymerase types. The solid-coloured lines denote the temporal trend of the top five major polymerase types per year. The pie charts indicate the prevalence of different genotypes and polymerase types detected before (left) and after (right) 1995. The dotted grey lines represent the total number of human norovirus sequences reported for each gene between the years 1995 and 2024.

**Fig. 2.**
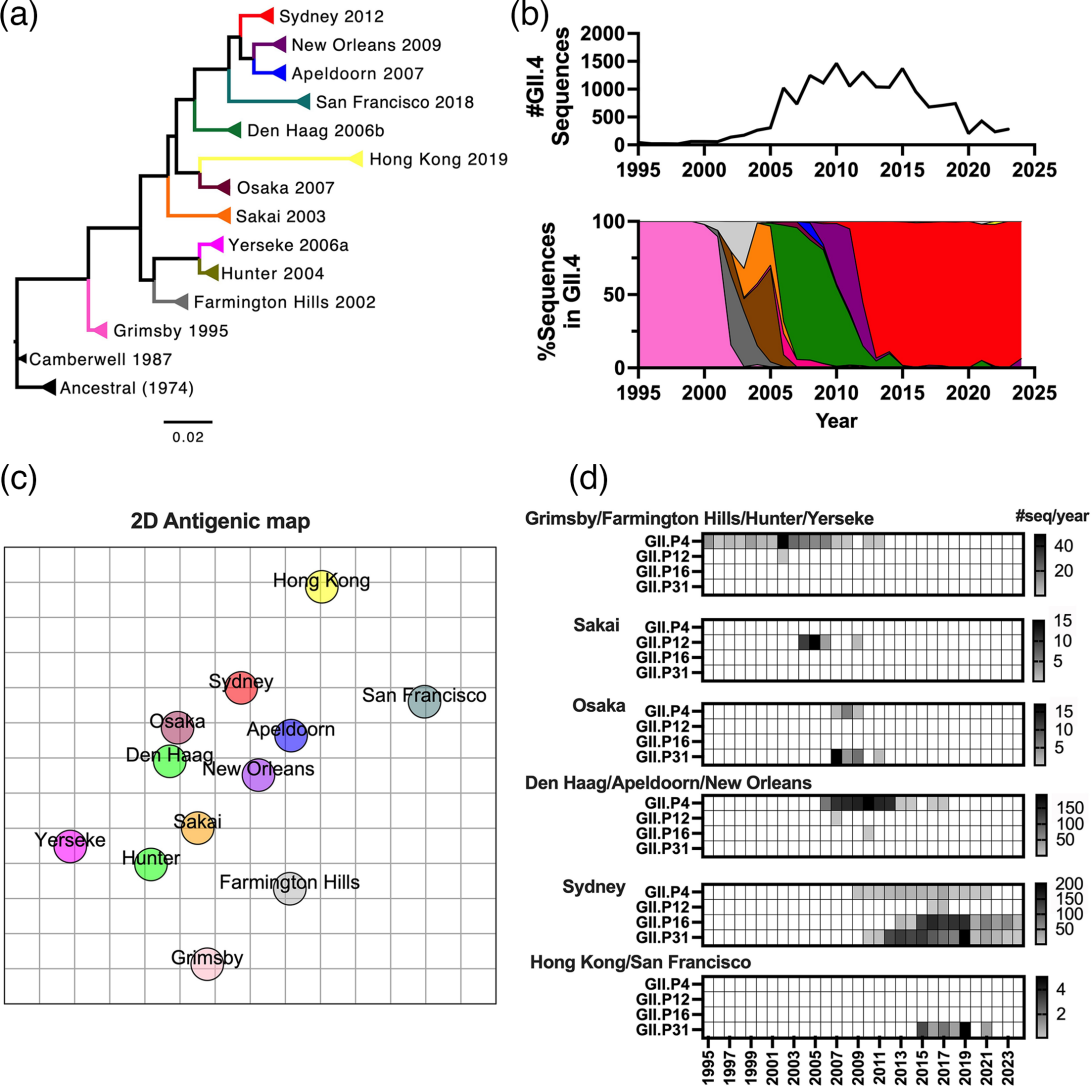
Evolution and characteristics of GII.4 norovirus variants that emerged since 1995. (**a**) Phylogenetic relationship of GII.4 variants inferred using VP1 amino acid sequences through a maximum likelihood method using mega v11 [111]. Each colour on the branch denotes a different GII.4 variants that emerged at different time points. Representative viruses from each variant were used to construct the tree [GenBank accessions: ancestral (JX023286), Camberwell (AY032605), Grimsby (AJ004864), Farmington Hills (DQ658413), Hunter (EU078414), Sakai (AB220922), Yerseke (EF126963), Den Haag (EF126965), Osaka (AB434770), Apeldoorn (AB541274), New Orleans (KX353958), Sydney (KY424328), Hong Kong (MN400355) and San Francisco (MW506849)]. (**b**) The top line graph indicates the number of GII.4 norovirus sequences reported between the years 1995–2024 (*n*=16,826). The bottom area plot indicates the temporal trend of GII.4 variants as represented by the ratio (%) per year. The colour of the area follows the colour palette of each variant in panel a. Classification of the GII.4 variants was determined by the Norovirus Typing Tool [110]. (**c**) Two-dimensional antigenic cartography of GII.4 variants confirmed the antigenic diversity among chronologically emerged GII.4 variants. Each unit of the grid represents a twofold difference of HBGA-blockade EC_50_ titers. The antigenic map was created using previously published blockade EC_50_ titers of sera collected from VLP-immunized mice [42,77] and the Racmacs package in R v4.2 [112]. (**d**) The heatmaps show the temporal prevalence of polymerase types from different GII.4 variants. Variants presenting the same polymerase types are grouped within the same heatmaps.

The first evidence of GII.4 predominance emerged during the molecular characterization of gastroenteritis outbreaks in southwest England in 1992, where most of the Norwalk-like viruses detected were identified as part of the Bristol (B493) virus cluster [30]. However, a retrospective study of several outbreaks in Maryland nursing homes during the winter season of 1987–1988 provided evidence for the widespread circulation of GII.4 noroviruses during the late 1980s [31]. In 1995, a new GII.4 virus (or variant), Grimsby 1995, emerged and predominated in the UK and USA as the main cause of acute gastroenteritis outbreaks [32]. Since then, GII.4 norovirus has prevailed among all the other genotypes globally (Fig. 1a) [33], and this prevalence was associated with the chronological emergence of several distinct variants (Fig. 2a, b). The Farmington Hills 2002 variant emerged 7 years later [34], and one distinctive characteristic of this variant was a single amino acid insertion in what is now recognized as one of the key antigenic sites in the VP1 [35]. Since 2002, a rapid turnover of new variants was observed: Hunter 2004, Yerseke 2006a, Den Haag 2006b, New Orleans 2009 and Sydney 2012 variants predominated globally, while Sakai 2003, Osaka 2007 and Apeldoorn 2008 variants presented limited incidence and distribution (Fig. 2b) [36]. This continuous emergence and succession of GII.4 variants was linked to mutations on VP1 that allowed the escape from immunity elicited by previous infections [37,41]. Indeed, reactivity with different antibodies and polyclonal sera from experimental animals and infected individuals confirmed the antigenic differences among these variants, as represented by positional differences on an antigenic map (Fig. 2c) [38,42,44]. These mutations have been mapped to at least five antigenic sites (A, C, D, E, G) on the surface of the capsid P domain [37,41].

Recombination, primarily within the ORF1/2 overlapping region, is another contributing factor to norovirus evolution and diversity [45]. In addition to the accumulation of mutations on the major capsid protein, GII.4 noroviruses have presented ORF1 sequences from different polymerase types (Fig. 2d). The GII.P4 polymerase, the prototype for GII.4 noroviruses, was first detected in viruses circulating in 1987 [46]. Meanwhile, GII.4 noroviruses circulating during the 1970s and early 1980s presented the GII.P39 polymerase [47,48]. Since the 1980s, the GII.P4 polymerase was almost exclusively associated with most variants up to the emergence of the New Orleans 2009 variant (Fig. 2d). Only a few sporadic cases of the virus presenting the GII.P12 and GII.P16 polymerase types were detected during that time. Two variants, Sakai 2003 and Osaka 2007, constituted the exception; these variants were mostly associated with the GII.P12 and GII.P31 polymerases, respectively (Fig. 2d). The change of ORF1 sequence seemed to have no impact on their transmissibility, as neither of the two variants predominated in the human population (Fig. 2b). A big change in this pattern was observed when the Sydney 2012 variant emerged. Although the ancestral and minor populations of Sydney 2012 viruses presented the prototypic GII.P4 polymerase, most viruses from this variant were reported with GII.P31 [49]. Notably, while the Osaka 2007 and Sydney 2012 variants shared the same polymerase type (GII.P31), only Sydney 2012 prevailed globally [48]. Afterwards, the Sydney 2012 variant acquired additional polymerase types designated GII.P12 and GII.P16, and the latter (GII.4 Sydney 2012[P16]) co-dominated with Sydney 2012 presenting the GII.P31 polymerase (GII.4 Sydney 2012[P31]) (Fig. 1b) [50].

## Episode III: the revenge of non-GII.4s

After a decade (2002–2012) of the frenetic emergence of several GII.4 variants, it became clear that other genotypes can temporarily become predominant and spread globally [51,52]. A notable example of this is the rapid rise of GII.17 and GII.2 cases during the 2013–2015 and 2016–2017 seasons, respectively (Fig. 1). GII.2 norovirus has been historically associated with multiple outbreaks, especially among the paediatric population [53,54], but the steep rise and predominance of GII.2 in the 2016–2017 season was observed in all age groups [55]. These changes in predominance seemed to be related to changes in the polymerase (or other ORF1 proteins) without apparent antigenic changes in VP1 [56,57]. Notably, these GII.2[P16] viruses presented an ORF1 sequence very similar to the ones presented by GII.4 Sydney 2012[P16] viruses circulating since 2015 [50,56]. On the other hand, the surge in GII.17 outbreaks, particularly in Asia, was linked to the emergence of a variant with a new capsid and a new polymerase type, GII.P17, that quickly spread across multiple countries on all continents (Fig. 1b) [58,59]. For almost four decades, GII.17 noroviruses were detected sporadically, with fewer than 100 sequences reported across 22 countries. However, by 2013, the number of GII.17 detections surged, with thousands of reports across more than 50 countries [51,52, 60, 61]. Since then, GII.17 has consistently ranked among the most common norovirus genotypes worldwide, but GII.17 infections (and the number of outbreaks) declined by 2020.

**Fig. 3.**
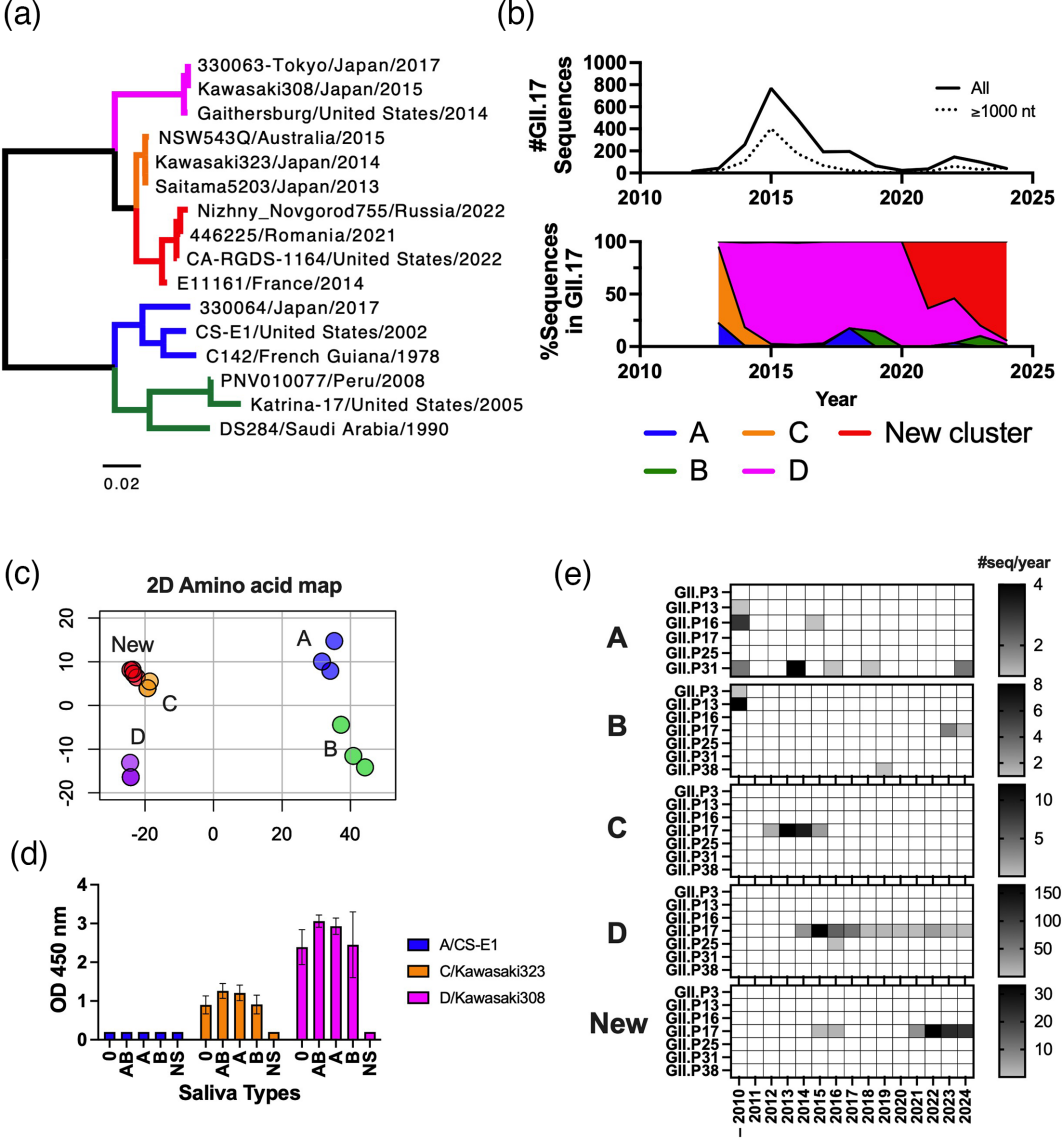
Evolution and characteristics of GII.17 norovirus variants detected in humans. (**a**) Phylogenetic relationship of GII.17 noroviruses inferred using VP1 amino acid sequences through a maximum-likelihood method using mega v11 [111]. Each colour on the branch denotes different clusters of GII.17 noroviruses; cluster A represented by CS-E1/2002 (blue), cluster B represented by Katrina/2005 (green), cluster C represented by Kawasaki323/2014 (orange), cluster D represented by Kawasaki308/2015 (purple) and a recently emerged new cluster represented by Romania/2021 (red). (**b**) The top solid line graph indicates the number of GII.17 norovirus sequences reported between the years 2010–2024 (*n*=2,381). As GII.17 clusters were not able to be appropriately classified using short partial genomes (e.g. ~300 nt of conserved shell domain), VP1 sequences with ≥1,000 nt were retrieved (*n*=974) and subjected to phylogenetic analysis to classify the viruses into the clusters. Thus, the dotted line in the graph summarizes the number of sequences with ≥1,000 nt, which reflects the overall temporal trend of GII.17 sequence reports as shown by the solid line. The bottom area plot indicates the temporal trend of GII.17 clusters as determined using ≥1,000 nt-length VP1 sequences. The colour of the area follows the colour palette of each cluster in panel A. (**c**) Multidimensional scaling analysis of VP1 amino acid sequences used in the tree (panel a) suggests different antigenic profiles of GII.17 clusters, while the newly emerged cluster was closely located to cluster C viruses with 10–15 amino acid mutations. (**d**) GII.17 clusters presented different HBGA-binding patterns as measured by ELISA. The binding data of VLPs and HBGA molecules in saliva from individuals with different blood types (O, A, B, AB, NS; non-secretor) were extracted from a previous study [72]. (**e**) The heatmaps show the temporal prevalence of polymerase types from different GII.17 clusters.

Genetic analyses of GII.17 viruses revealed the presence of at least four distinct phylogenetic clusters (A–D) (Fig. 3a) [61,62]. Early data suggested that cluster A viruses circulated in the 1970s, cluster B viruses from 2005 to 2009, cluster C viruses from 2013 to 2014 and cluster D viruses since 2013. Thus, the initial interpretation was that this genotype, similarly to GII.4, exhibited periodic emergence and replacement of discrete phylogenetic clusters (or variants) [62,63]. However, with the increasing availability of sequence data, it became clear that variants A and B have been circulating continuously at low levels in the human population for over 30 years (Fig. 3a, b) [48]. Despite this low circulation, outbreaks linked to variant A or B viruses have been described [64,66]. The origin of cluster C and D viruses is still a subject of debate. One study proposed that they originated from viruses circulating in Africa in 2001 [59]. However, ancestral viruses from these clusters, such as Tokyo/27-3/1976 (cluster C), have been detected as far back as the 1970s [67]. Thus, it is very likely that viruses from the same lineage of cluster C and D have also been continuously circulating in humans. While cluster D viruses have spread rapidly across continents, analysis of complete VP1 sequences indicated that they likely originated from a single haplotype that circulated in China in late 2014 [59,68].

These GII.17 variants differ by ~25–72 (4.5–13.2 %) aa in the VP1 sequences (Fig. 3c). Most of these differences map to the P domain of VP1. Sequence analysis of the VP1 region from viruses representing each cluster revealed several amino acid substitutions unique to each variant. For example, variant D viruses exhibited three deletions (residues 295, 296 and 384) and two insertions (I375 and D396) compared to variants A and B and 25 amino acid substitutions, including two insertions (I375 and D396), when compared to variant C viruses. Variant B viruses showed several unique substitutions, including a single insertion (G344), as compared to variant A viruses. The two-amino acid deletion (295–296) in variant D viruses mapped to the same immunodominant region (antigenic site A) described for GII.4 norovirus. The remaining insertions and deletions detected across the GII.17 variants were also situated near regions of VP1 known to correspond to major antigenic sites in GII.4 noroviruses. Several studies using human and animal sera, as well as monoclonal antibodies (mAbs), have shown that viruses from variants A, B and D are antigenically distinct [63,69]. However, the specific substitutions responsible for these differences remain undetermined. One study, which characterized four mAbs and virus-like particles (VLPs) from variants B, C and D, found that the region spanning aa 293–299 is critical for the binding of two blocking mAbs that exhibit variant specificity [70]. It remains to be determined whether this is an immunodominant region as was described for GII.4 noroviruses.

In addition to presenting differences in antigenicity, the emergence and predominance of GII.17 viruses during 2013–2015 were also associated with changes in their ability to infect different populations [71,73]. Thus, it has been shown by at least three independent groups that viruses from GII.17 cluster D present an enhanced binding to HBGAs as compared to previously circulating GII.17 cluster A and B viruses (Fig. 3d). Another notable aspect of the evolution of GII.17 variants is their specific association with different ORF1 regions. Variant A viruses are linked almost exclusively with GII.P16 and GII.P31 polymerase types, variant B viruses to GII.P13 and variants C and D to GII.P17, a polymerase type first identified following the emergence of these two variants (Fig. 3e) [60,61]. A small number of recombinant GII.17 viruses were detected with GII.P3 (*n*=1), GII.P25 (*n*=3) and GII.P38 (*n*=1). All these recombinant forms are in accordance with the recombination patterns described for all human noroviruses [48]. Together, changes in the antigenicity, NS proteins and an enhanced binding to HBGA molecules might have played a role in the high incidence of GII.17 viruses during 2013–2015.

## Episode IV: the return of GII.4

With the emergence and high incidence of GII.17 viruses during 2013–2015, it was suggested that the dominance of GII.4 could have reached an end [74]. This hypothesis was further supported by the transient dominance of GII.2 viruses during 2016–2017; however, by 2019, GII.4 Sydney 2012 returned as the predominant norovirus genotype in multiple countries (Figs1  2) [51]. This predominance was attributed to (i) the co-circulation and co-dominance of phylogenetic lineages represented by two recombinant forms: the original Sydney 2012[P31] and a new recombinant Sydney 2012[P16] and/or (ii) compromised immune responses in adults that facilitate the continued circulation of this variant [43]. The emergence of Sydney 2012[P16] was not associated with a major increase in the number of outbreaks reported worldwide [50,75]. Despite presenting distinct lineages, the VP1 of all Sydney 2012 viruses presented clock-like linear evolution and multiple amino acid differences that did not result in major antigenic changes [76]. Since the emergence of Sydney 2012, no turnover of new variants was reported; however, two genetically distinct clusters of viruses, Hong Kong 2019 and San Francisco 2017 [77,80], were reported to be circulating since 2016. These two variants are antigenically distinct from Sydney 2012 viruses [43,77]. The reason why these two variants did not displace variant Sydney 2012 as predominant remains to be determined.

## Episode V: GII.17 strikes back

After a period of relative calm, a large number of norovirus outbreaks were reported in Europe and North America during 2023–2024 [81,83]. Surprisingly, GII.17 viruses were detected as the main cause of these outbreaks, but even more surprising was that these viruses were related to the cluster C lineage (Fig. 3a). Viruses from cluster C were thought to have died out and been replaced by the cluster D viruses; however, these viruses were detected sporadically in France and Canada in 2014 and 2015, respectively (Fig. 3a) [81]. Genetic characterization of these new emerging ‘cluster C’ viruses identified multiple amino acid mutations at the P domain as compared with previously circulating GII.17 viruses [82,84] (Fig. 3c). In addition, while their ORF1 region was typed as GII.P17, the non-structural proteins from these new viruses were phylogenetically separated from those of GII.P17 in the clusters C and D [81,82]. It is also noteworthy that GII.17 viruses from cluster B were also reported in the USA in 2023 and 2024 [81]. Only four viruses have been reported in GenBank so far, but interestingly, their ORF1 is typed as GII.P17, the same polymerase type shared with the predominant cluster C, D and new GII.17 viruses (Fig. 3e). Further studies are warranted to observe if the recent cluster B viruses could benefit from the ORF1 pandemic GII.P17 polymerase type. It remains to be determined whether the re-emergence of cluster C (and potentially cluster B) viruses is associated with (i) new antigenic characteristics that can evade immune responses developed against previous GII.17 clusters, (ii) a differential HBGA binding profile that facilitates infection of susceptible populations, (iii) better transmissibility due to the mutations on the nonstructural proteins or (iv) a combination of the above (or other) characteristics that led to its re-emergence.

## Episode VI: a new hope

**Fig. 4.**
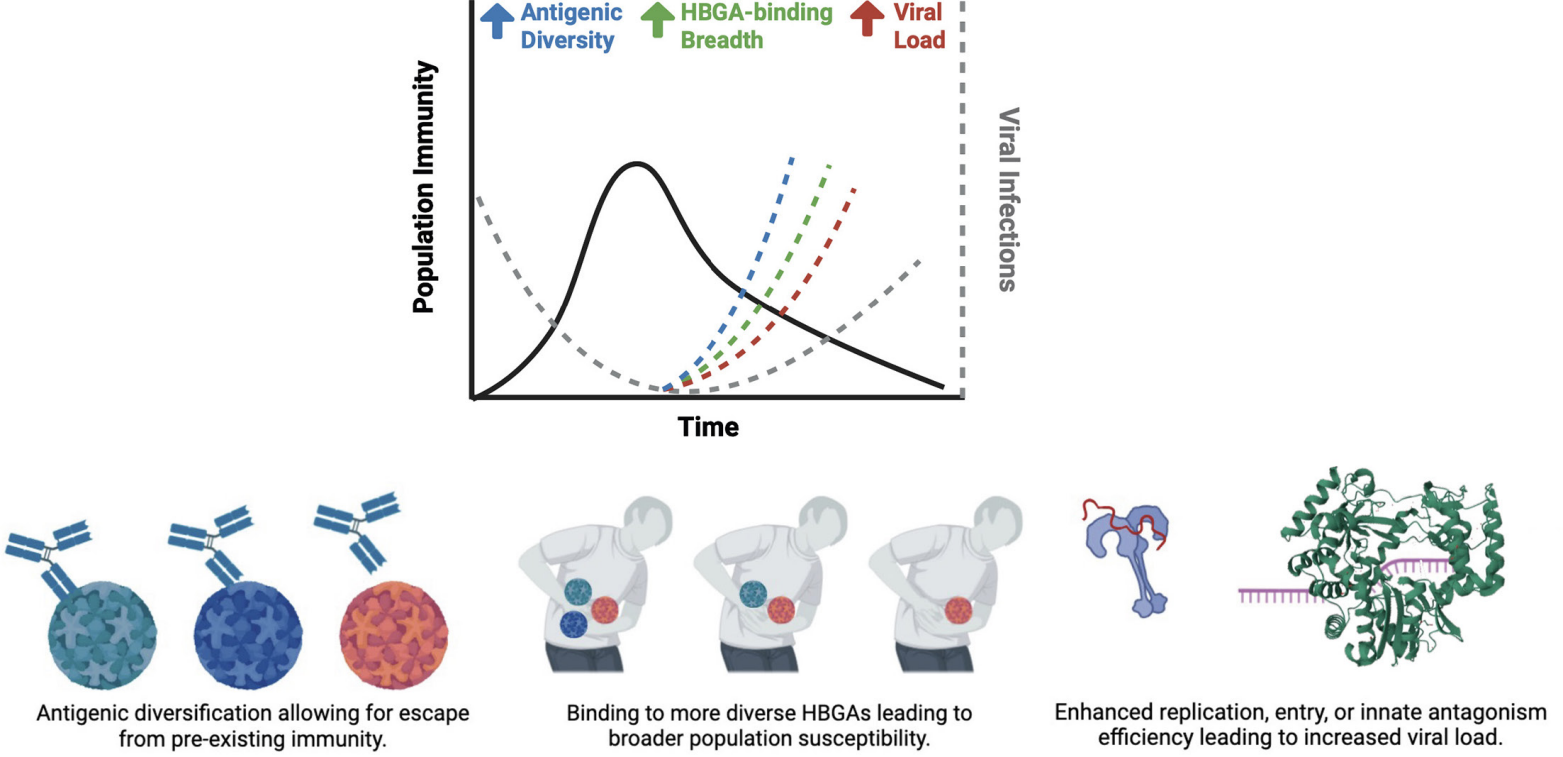
Norovirus outbreaks are likely driven by a combination of host and viral factors. Following initial infections, the population develops immunity (indicated by the black solid line), leading to a decline in subsequent infections over time and contributing to herd immunity. However, the number of viral infections in the population (indicated by the grey dotted line) could be influenced by factors such as the birth of new individuals (or the replenishment of a naïve population) or the waning of antibody responses from primary infections. To accelerate reinfection rates, norovirus may (**i**) undergo antigenic diversification, allowing it to escape pre-existing immunity; (ii) alter its binding profile to HBGA attachment factors, increasing susceptibility across a broader population; and (iii) enhance viral load through mechanisms such as increased RNA replication, improved entry or innate immune antagonism. These factors – whether individually or in combination – can drive the emergence of new strains and accelerate reinfection, even in the presence of prior immunity.

The recent surge of GII.17 cases in Europe, Japan, and the USA [81,85] has reinforced the impact of norovirus on human health. Despite its diversity, relatively few norovirus genotypes make up the majority of infections every year, and even fewer (such as GII.4, GII.2 and GII.17) are responsible for large-scale outbreaks [33,50, 52, 57, 58, 68, 81]. It is unclear what makes these predominant genotypes special or whether it is the convergence of both host and virus factors that allows outbreaks to occur. Epidemiological and human challenge studies have revealed that infection provides relatively short-term immunity against the infecting agent and offers little protection against heterologous genotypes [15,16, 86,89]. Immunity to norovirus gastroenteritis has been estimated to last approximately 4 years [90]; therefore, waning population immunity could then begin to explain these periodic outbreaks. However, the fast-evolving nature of norovirus further contributes to immune escape of any maintained protective responses [39,41, 91]. Thus, the interplay between waning immune responses and antigenic diversification could together facilitate reinfections and large-scale outbreaks. Susceptibility to norovirus disease is determined by expression of HBGAs, and norovirus genotypes that can use a broader range of host carbohydrates could also likely contribute to outbreak potential. Further, mutations acquired in the RNA polymerase or non-structural proteins could increase replication efficiency and transmissibility [92,93]. Therefore, it is likely that a combination of some or all these factors creates the perfect scenario for norovirus outbreaks (Fig. 4).

Norovirus disease is associated with up to 200,000 deaths annually and is estimated to cost society $60 billion every year [94,95], but despite this high burden, no prophylactic or virus-specific therapeutic treatment is available. The development of countermeasures has been historically impaired by several factors, including the lack of a robust cell culture or animal disease model and marked viral genetic and antigenic diversity. However, recent breakthroughs have provided new hope towards the development of broadly protective agents for norovirus. The development of a human intestinal enteroid culture system has propelled our ability to interrogate the viral lifecycle and provides a more direct mechanism to measure viral neutralization and its correlation with HBGA blockade titers [44,96, 97] – a key correlate of protection signal that could be implemented in large clinical trials [98,100]. Further, the establishment of a non-human primate model of norovirus infection has already begun to provide critical insights and will allow for improved preclinical testing of vaccines or antivirals [101]. In addition, despite the extensive diversity posed by norovirus, several promising vaccine candidates have entered clinical trials. Vaccine strategies include protein VLPs, adenovirus-vectored antigens and mRNA-encoded approaches [98,102, 103]. As GII.4 has historically shown predominance for decades, multiple approaches have focused on GII.4 antigens, but clinical trials with multi-valent strategies, including different combinations of genotypes, have begun in the past few years [98,100]. Encouragingly, multiple studies have now reported monoclonal antibodies and human serum samples with cross-neutralizing activity, suggesting that broadly therapeutic antibodies can be developed, and protective immunity can be elicited [44,104,108]. Furthermore, a recent vaccine trial reported protection from norovirus disease by a non-vaccine matched genotype, providing field evidence that broad immunity can be achieved [102,109].

Considering both viral and host factors, including genetic and antigenic diversity, HBGA susceptibility, transmission efficiency and population immunity, we revised the model for how the emergence of predominant viruses may occur (Fig. 4). While this virus continues to surprise us, both clinical and pre-clinical advancements offer new hope for the development of effective prophylactic and therapeutic interventions for human norovirus.

## Abbreviations

FUT2: fucosyltransferase 2
HBGA: histo-blood group antigen
mAbs: monoclonal antibodies
VLPs: virus-like particles
